# Tungsten and Molybdenum Heteropolyanions with Different Central Ions—Correlation between Theory and Experiment

**DOI:** 10.3390/molecules27010187

**Published:** 2021-12-29

**Authors:** Piotr Niemiec, Renata Tokarz-Sobieraj, Małgorzata Witko

**Affiliations:** 1 Faculty of Mathematics and Natural Sciences, Department of Chemistry, University of Applied Sciences in Tarnow, Mickiewicza 8, 33-100 Tarnow, Poland; 2 Jerzy Haber Institute of Catalysis and Surface Chemistry, Polish Academy of Sciences, Niezapominajek 8, 30-239 Krakow, Poland; malgorzata.witko@ikifp.edu.pl

**Keywords:** heteropolyacids, DFT calculations, correlations analysis, redox potential, energy decomposition analysis (EDA)

## Abstract

Density functional theory calculations were carried out to investigate the electronic structures of Keggin-typed [XMo_12_O_40_]^n−^ and [XW_12_O_40_]^n−^ anions with different heteroatoms (X = Zn^2+^, B^3+^, Al^3+^, Ga^3+^, Si^4+^, Ge^4+^, P^5+^, As^5+^, and S^6+^). The influence of solvent on redox properties of heteropolyanions was discussed. For [XW_12_O_40_]^n−^ systems two linear correlation: first, between the experimental redox potential and energies of LUMO orbital; and second, between the experimental redox potential and total energy interaction (calculated between internal tetrahedron (XO_4_^n−^), and rest of Kegging anion skeleton, (W_12_O_36_)) were designated. Taking into account the similarity of XW_12_O_40_^n−^ and XMo_12_O_40_^n−^ systems (in geometry and electronic structure), the estimated redox potential of molybdenum heteropolyanions (with X being p block elements) in different solvent were proposed.

## 1. Introduction

Heteropolyacid (HPA) systems belong to a group of compounds, which due to their acid–base and redox properties, are used in numerous catalytic processes both homogeneous and heterogeneous [1,2,3,4,5,6,7,8,9,10,11,12,13,14,15,16]. They have several advantages as catalysts, which make them economically and environmentally attractive; the most meaningful are their versatility (in the sense of electronic and geometry structure as well as chemical composition), performance in moderate reaction conditions, thermal stability and stability in solutions, simple recovery, and recycling. HPA catalysts are currently used in several industrial processes including selective oxidation of ethene/methacrolein, hydration of propene/buthene, polymerization of tetrahydrofuran and coupling reactions [3].

The important parameter that makes HPAs useful in catalysis is their reduction potential (oxidizing power) measuring of tendency of chemical species to acquire electrons, and thereby become reduced. Several theoretical and experimental methods were employed to determine that parameter. The reduction potential of HPA can be established by the measuring of absorption edge positions in the UV–Vis spectra [17] or negative differential resistance (NDR) peak voltages in scanning tunneling microscope STM [17,18,19,20,21,22], but the most conventional technique is the electrochemical method. Redox potential obtained by electrochemical measuring depends on composition of electrolyte solution (pH), the identity of the electrodes and other parameters (for example, measurement conditions). As a result, there is little experimental data obtained in the same condition for various solvent and different HPA [23,24,25]. Although, there are some exceptions including: redox potential for H_n_XMo_12_O_40_ (where: X = P, As, Si, Ge) [26], for H_n_XW_12_O_40_ (where X = P, Si, Co, Fe, B) [23,25,27,28,29], modified molybdenum and tungsten heteropolyacids with P^5+^/Si^4+^ as central ion in various solvents, such as H_2_SO_4_ [30], HClO_4_ [31], CH_3_CN [32], and Na_2_SO_4_ [33]. In all of the abovementioned papers/cases, the correlation between one-electron redox potential and global ionic charge of heteropolyacids systems were reported.

Several theoretical methods were employed to determine the theoretical description of redox potential in HPA systems [26,34,35,36,37,38]. This matter was addressed mainly by Poblet group [34,35,36,37] who studied the XM_12_O_40_^n−^ and SiM_11_VO_40_^5−^ systems (M = W^6+^, Mo^6+^, X = Al^3+^, Si^4+^, P^5+^, Fe^3+^, Co^3+^, and Co^2+^) and demonstrated that the introduction of additional electron to the system results in its delocalization onto all twelve poly-atoms and formation of reduced “blue species.” An attempt to systematize the assessment of the oxidation and reduction abilities of heteropolyanions was undertaken in [35,36] through analysis of the so-called anion charge effect. It was shown that by defining the molecular charge density *q*/*m* (*q* = total charge of heteropolyanion, *m* = number of equivalent metal centers) the results can be generalized to all polyoxometalates. This conclusion was confirmed by Pope in 1983 [36], who assumed that the reduction potential changes by −0.18 V per each unit of the total heteropolyanion charge with the Keggin structure. The influence of the size of the central ion on the redox properties of HPAs systems was examined in [38] where it was demonstrated that the chemical nature of central ion determines the electrostatic potential of the internal tetrahedron, affecting the metal and oxygen skeleton. Central units with a greater negative charge (e.g., −6, XO_4_^n−^) achieve more negative potential in the metal–oxygen environment W_12_O_36_, which reduces the system’s tendency to receive electrons, lowering the redox potential. Attempts to correlate redox properties with the atomic radius of the central ion (the size of the internal tetrahedron) evidence that the smaller the tetrahedron size and the larger its charge, the greater impact on the metal–oxygen skeleton. Another factor that affects redox property of HPA system is its geometric structure [34]. Based on DFT calculations carried out for the five geometric isomers of the Keggin anion it was found that the mutual arrangement of triplet units determines the energy of unoccupied frontier orbitals affecting oxidation and reduction abilities of the systems that change in order: α > β > γ > δ > ε.

For a long time, HPA science was concentrated mainly around designing and synthesizing structures but for some time it is gravitating towards the examination of more interdisciplinary multifunctional materials and related areas to diversify future possibilities of POMs (Polyoxometalates) [38,39]. Thanks to the attractive topologies and exceptional properties explored in gas adsorption and separation, heterogeneous catalysis, ion exchange, chiral resolution, magnetism, luminescence, and nonlinear optical properties POM scientists have taken up a challenge to introduce POMs into Coordination Polymer systems (CP) building POMCPs. CPs are built from metal ions or metal clusters and organic ligands unit joined together via coordination bonds (or even supramolecular interactions); they present a highly ordered structure with repeating coordination entities extending in one, two, or three dimensions [40]. Keggin-type POMs, can be treated as one of the best candidates for designing functional POMCPs. Furthermore, POMCPs based on Keggin-type unit represent over 50% of the described members of this family [41,42]. In addition, one should stress that POMCPs present a lot of different and attractive applications in: photocatalysis [43,44,45,46,47,48,49,50,51,52,53,54,55,56,57,58,59], electrocatalysis [60,61,62,63,64,65,66,67,68], magnetism [69,70,71,72,73,74], and photoluminescence [75,76,77,78,79,80,81,82,83].

The main goal of this work is to find the correlation between redox potentials and calculated parameters describing the electronic structure of modified HPA by discussing the impact of various central ions on the electronic structure of [XMo_12_O_40_]^n−^ and [XW_12_O_40_]^n−^ Keggin anions.

## 2. Results and Discussion

From the literature data it is known that redox potential of heteropolyacids systems depend on the global charge of the heteropolyanion; there is no further information about differences between systems with various central ions having the same global charge. Therefore, we decided to analyze and find parameters, which not only determine the electronic structure of HPA systems with various central anion, but also can be compared with experimental data.

### 2.1. XW_12_O_40_^n−^ Systems

The available literature data suggest that the search for a quantitative correlation between the experimental results and the calculated theoretical parameters should be primarily focused on the analysis of boundary orbitals. Therefore Table 1 summarizes the energy of frontier orbitals (HOMO, LUMO) and the size of the band gap, for XW_12_O_40_^n−^ systems for calculations made in vacuum and in solvents: acetonitrile and water as generally used in the reaction with heteropolyacids systems. The presented results show the large differences for systems under vacuum and in solution. In a vacuum, energies of frontier orbitals for various systems strongly depend on X belonging to particular groups in the periodic table. The differences in values of energy of frontier orbitals between groups can be as high as 2–3 eV (e.g., energy of LUMO orbital in vacuum: Zn^2+^ 10.52; B^3+^ 7.99; Si^4+^ 5.17). Much smaller differences are observed for the systems tested in solvents. Here on going from group to group, energies of HOMO/LUMO orbitals changes about 0.3 eV (e.g., energy of LUMO orbital in water: Zn^2+^ −3.55; B^3+^ −3.77; Si^4+^ −4.14). Within group (for cations with the same valence charge) the energies values of frontier orbitals are much smaller (e.g., energy of HOMO orbital in water: Si^4+^ −6.74; Ge^4+^ −6.73).

**Figure 1:**
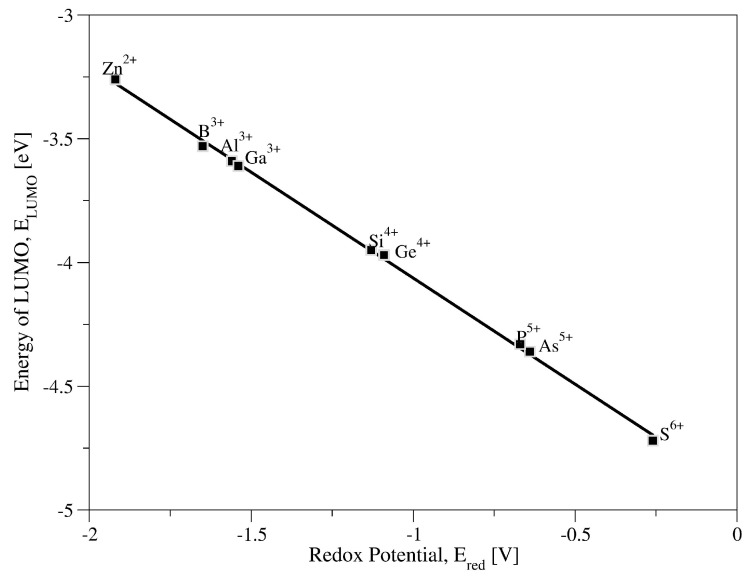
Correlation between energy of LUMO (Lowest Unoccupied Molecular Orbitals), E_LUMO_ (eV), and experimentally obtained [84] redox potential, E_red_ (V), for XW_12_O_40_^n−^ in CH_3_CN as a solution.

The theoretical parameters calculated for anions system in acetonitrile such as the energy of the HOMO or LUMO orbitals and the size of the energy gap are confronted against redox potential, obtained experimentally by K. Nakijima [84] through performing cyclic voltamperometry measurements (water content below <50 ppm). Only the correlation of the LUMO energy with the redox potential values is linear. The results presented in Figure 1 shows the linear correlation (R^2^ = 0.999) between calculated energy of LUMO orbital, E_LUMO_, and experimental values of the redox potential E_red_. From correlation it is evident that increase in LUMO energy causes decreases in redox potential, and as a result, oxidizing power of heteropolyanion weakens. Based on LUMO energy levels for two boundary systems, with the highest (ZnW_12_O_40_^6−^) and the lowest anion charge (SW_12_O_40_^2−^), −3.26 eV and −4.72 eV, respectively, one can indicate the SW_12_O_40_^2−^ system as exhibiting the highest oxidizing power, whereas ZnW_12_O_40_^2−^ as showing the lowest one.

The correlations found for the studied systems call for a much closer analysis of the frontier orbitals. Table 2 shows the percentage composition of HOMO, LUMO orbitals of [XW_12_O_40_]^n−^ where X = Zn^2+^, B^3+^, Al^3+^, Ga^3+^, Si^4+^, Ge^4+^, P^5+^, As^5+^, and S^6+^. HOMO orbitals of the studied systems are almost identical except systems with B^3+^ and Zn^2+^ as the central ions whereas energies differ significantly. In all cases HOMO are composed of the *2p* orbitals of bridging oxygens (double-coordinated to two addenda atoms) Ob (43–47%) and Oc (50–52%). As previously noted, the exception is the system with B^3+^, in the position of the central ion, where HOMO is built of *2p* orbitals of internal, Oa, oxygen atoms, (∼77%), coordinated with the central ion and one addenda and *2p* orbitals of terminal oxygen ions Od (∼10%), individually coordinated with tungsten atoms. In case of second exception ZnW system, the orbital is composed of *3d* central ion, Zn^2+^ as well as of *2p* orbitals of oxygen ions from the internal tetrahedral Oa (∼51%) and orbitals of bridging oxygen ions Oc (∼26%) with admixture of *2p* Zn orbitals. Unlike the HOMO orbitals, the composition of the LUMO orbitals is identical for all calculated systems. Their consist of *5d* orbitals of addenda atoms W (∼75%) with small amount of *2p* orbitals of bridging oxygen centers Ob (∼12%) and Oc (∼13%).

**Figure 2:**
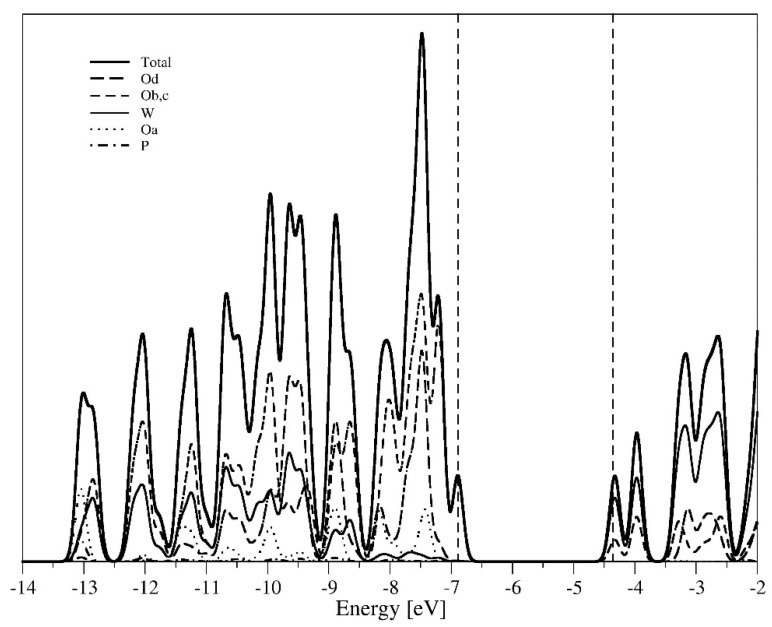
Total and partial density of states for PW_12_O_40_^3−^.

The received, almost identical, compositions of frontier orbitals do not explain neither the differences in the energy of the frontier orbitals nor differences in redox potential of the systems observed in the experiment. Therefore, the discussion was extended to the analysis of the valence band and the conduction band near the Fermi level. Figure 2 shows an example of the spectrum of electron density for the reference PW system. Analysis of various parts of the valence band and conduction band shows that in the valence band, in the immediate vicinity of the gap (located between the valence band and the conduction band) the bridging oxygens of Ob and Oc prevail, while in the area with slightly lower energy, the Od terminal oxygen ions dominate, next to which, there are also orbitals of internal oxygen ions Oa. In the central part of the valence band there are *2p* orbitals of mostly bridging and terminal oxygen ions with the insignificant amount of *5d* tungsten orbitals. The conduction band is built mainly of *5d* tungsten orbitals with a small amount of *2p* orbitals of bridging oxygen ions.

**Figure 3:**
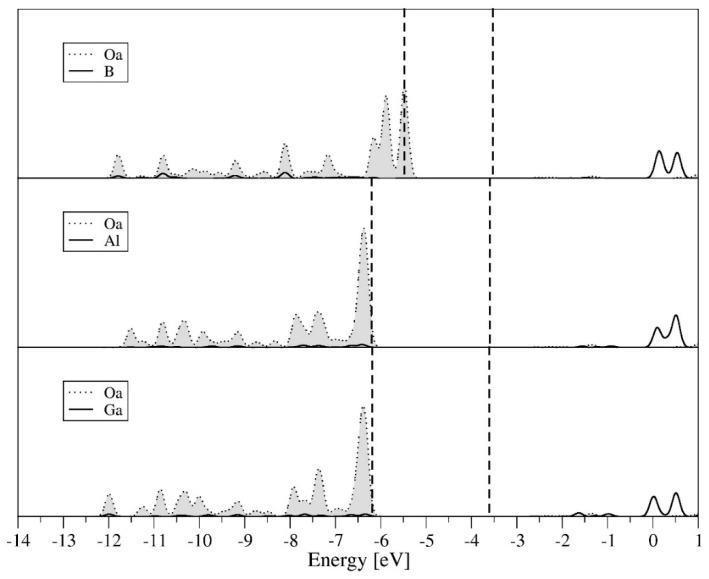
Partial (Oa and X atomic) density of states for XW_12_O_40_^3−^, where X = B^3+^, Al^3+^, Ga^3+^.

Figure 3 summarizes spectra of the partial density of states (coming from Oa and central ions orbitals) for systems having the same formal oxidation state of 3+ and belonging to the same third group of the periodic table. The analysis of the spectra leads to the conclusion that Oa orbitals are included only in the valence band, whereas the conduction band consist mainly of the orbitals of central ions. The participation of Oa orbitals in valence band is similar for all three elements of B, Al, and Ga, larger near the Fermi level and decreasing with the transition to areas with lower energy. The partial atom spectra of Oa oxygen ions coordinated with B^3+^ is slightly different from the image of Al and B, which applies both to the shape and position (*2p* orbitals of Oa oxygen ions in the BW system are located the closest to the Fermi level).

**Figure 4:**
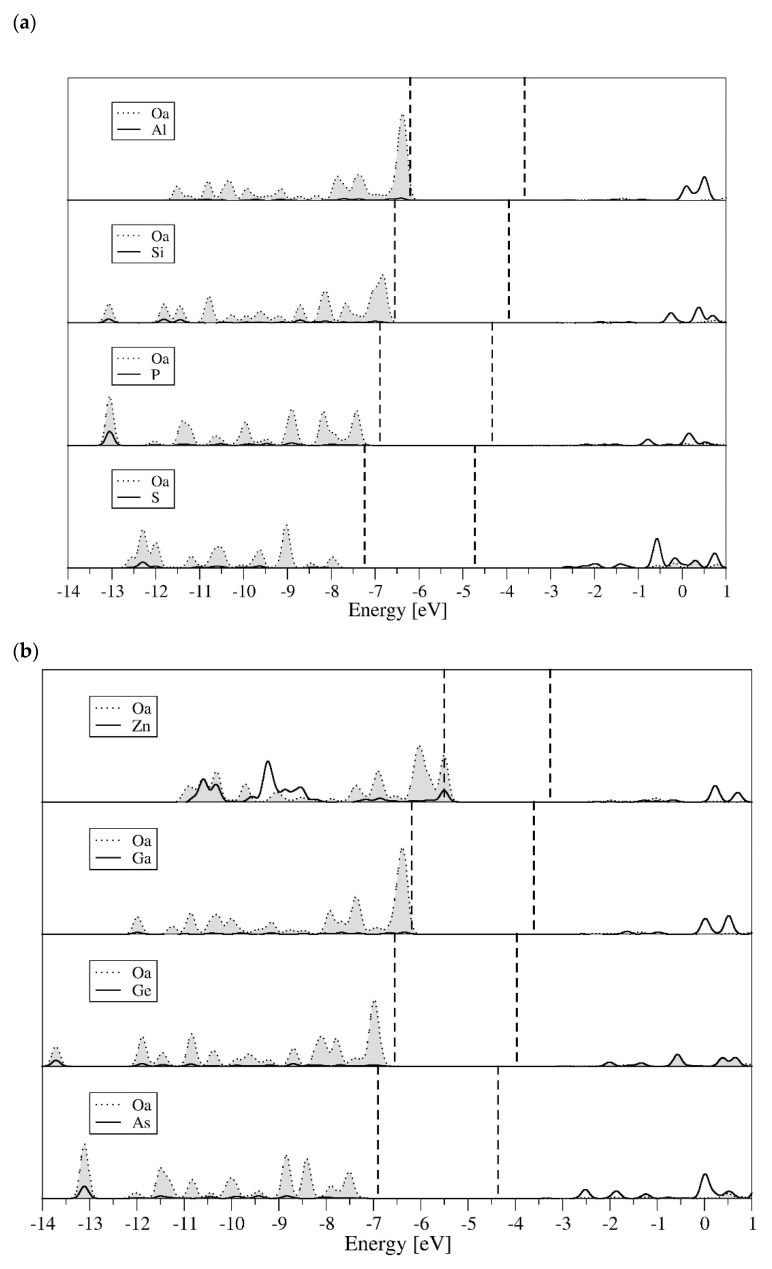
Partial (Oa and X atomic) density of states for systems with central ion belonging to third (**a**) and fourth (**b**) row of periodic table.

Comparison of the elements that belong to the same, third and fourth periods, respectively, which are presented in Figure 4a,b, show a strong variation in the position and the participation of orbitals coming from central ions and internal Oa oxygen ions. With the increase in electronegativity of the central ions (moving from Al to S in the third period and from Zn to As in the fourth period), the levels of frontier orbitals are shifting towards lower energies. Major changes are observed in the partial atomic spectra of oxygen ions coordinated with central ions. The partial density of states corresponding to oxygen ions coordinated with central ions decreases with increasing electronegativity of these ions (the transition from left to right of the periodic table). In the case of Al or Ga the part matching Oa oxygen ions is strongly visible in the vicinity of the Fermi level and is declining in areas with lower energy; while for the oxygen ions coordinated with S or As, it is almost identical throughout the whole area of the valence band. Partial density of states, belonging to the central ions, which appear in the conduction band increases with the increase in elements electronegativity (particularly visible for S and As). Zn is an element in which atomic spectrum is completely different from the other elements. *3d* orbitals of Zn central ion, and *2p* orbitals from the internal Oa oxygen centers, are strongly present in the valence band.

**Figure 5:**
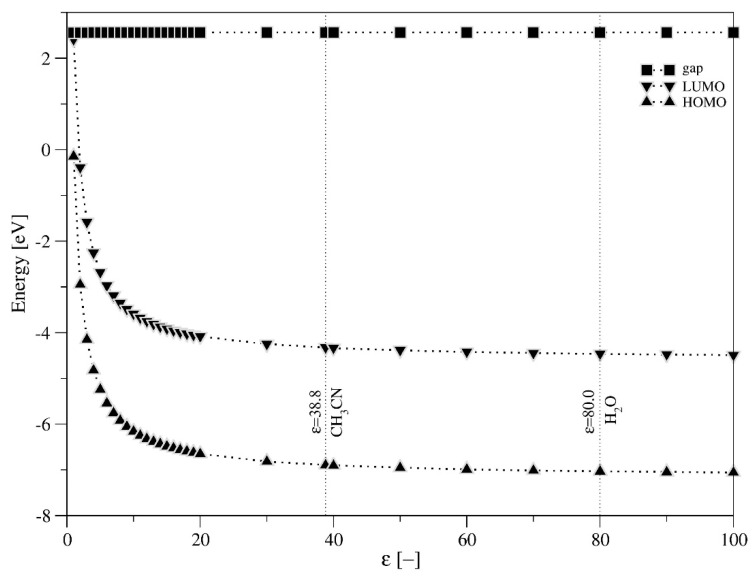
The relationship between values of dielectric constant and theoretically obtained energies of frontier orbitals and of band gap size for PW_12_O_40_^3−^ as reference system. Typical solvents used for HPA acetonitrile (εCH_3_CN = 38.8), water (εH_2_O = 80.0), acetone (ε(CH_3_)_2_CO = 20.7), methanol (εCH_3_OH = 32.7), and DMSO (ε(CH_3_)_2_SO = 46.7) are marked.

Data collected in Table 1, obtained for two solvents (acetonitrile and water), indicate that the reaction environment has a significant impact on the energy of HOMO and LUMO orbital. Extending the investigation to a wider range of solvents with dielectric constant, changing in the range from 1 to 100 (εH_2_O = 80, εCH_3_CN = 38.8, ε (CH_3_)_2_CO = 20.7, εCH_3_OH = 32.7, ε(CH_3_)_2_SO = 46.7) confirms the influence of the solvent on the energy of frontier orbitals and clarifies the need of the solvent effect to be considered in theoretical calculations. Figure 5 shows the theoretically obtained energy of frontier orbitals (HOMO, LUMO) and the size of the energy band gap versus the value of the dielectric constant (ε), characterizing the selected solvent, for the reference system PW_12_O_40_^3−^. From the analysis of Figure 5, one can conclude that both HOMO and LUMO energies decrease with increasing dielectric constant (ε). Changes are particularly evident in the interval of the dielectric constant (ε) from 1 to 20, whereas after exceeding ε = 20, discussed energies change insignificantly.

**Figure 6:**
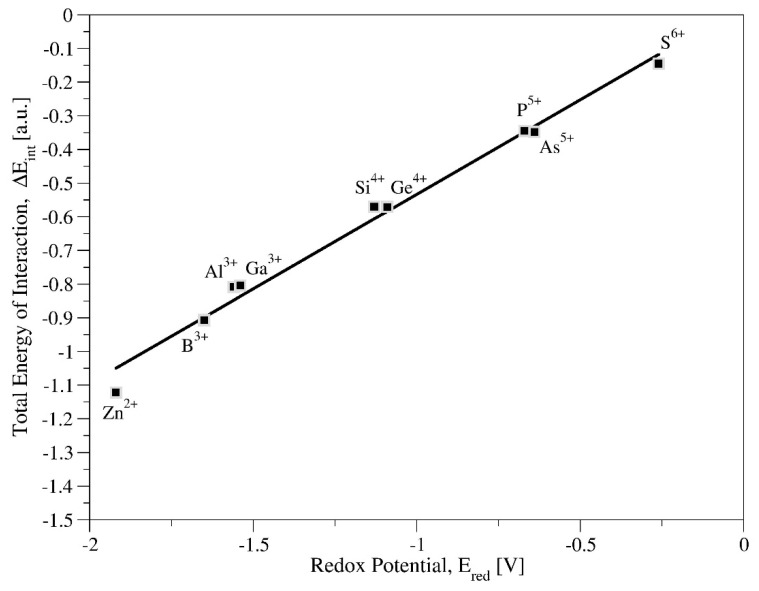
Correlation between total interaction energy ΔE_int_ (a.u.) and experimentally obtained [84] redox potential E_red_ (V), for XW_12_O_40_^n−^ in CH_3_CN as a solution.

The detailed analysis of the orbitals in the valence band, presented above, together with the indication of the participation of orbitals of both: the central ion X and coordinated with them orbitals of Oa oxygen atoms, prompted us to determine an additional theoretical parameter that distinguish these two (X, Oa) elements. Based on the geometric structure of the Keggin anion, in which the internal tetrahedron, XO_4_^n^ is surrounded by metal–oxygen skeleton W_12_O_36_, the interaction energy between these two building elements was determined. By applying the Energy Decomposition Analysis, the total interaction energy between the internal tetrahedron (XO_4_^n^) and the metal–oxygen framework (W_12_O_36_) was taken into consideration. Due to the lack of covalent bond (previous analysis of bond orders) between the internal tetrahedron (XO_4_^n−^) and metal–oxygen framework (W_12_O_36_), only the total interaction energy (ΔE_int_) was used, without taking into account its individual contributions. The calculations were made taking into account the solvation correction with ε = 38.8 in CH_3_CN, for which experimental redox potential values are available. The results are summarized in Table 1 in the penultimate row. The collected values show the dependence of the determined values of E_int_ on the substituted heteroatom X. Moving from Zn^2+^ to S^6+^, the energy of the total interaction varies from −1.122 a.u. to −0.145, respectively. Calculated values of total energy interaction were compared with values of the redox potential obtained experimental [84]. From correlation presented in Figure 6, it is evident that the increase in ΔE_int_ energy simultaneously causes increase in redox potential; as a result, oxidizing power of heteropolyanion strengthens. Based on ΔE_int_ for two boundary systems, with the highest (ZnW_12_O_40_^6−^) and the lowest anion charge (SW_12_O_40_^2−^), −1.122 a.u. and −0.145 a.u., respectively, one can conclude that SW_12_O_40_^2−^ system shows the highest oxidizing power, whereas ZnW_12_O_40_^6−^ the weakest one. The results presented in Figure 6 that show a linear correlation (R^2^ = 0.987) between both parameters, allows to define another, new theoretical parameter, which allows to quantify the redox potential of systems with a different central ion.

### 2.2. XMo_12_O_40_^n−^ Systems

Similar type of calculations was carried out for molybdenum heteropolyanions XMo_12_O_40_^n−^ modified in position of heteroatom by various elements X = Zn^2+^, B^3+^, Al^3+^, Ga^3+^, Si^4+^, Ge^4+^, P^5+^, As^5+^, and S^6+^. Table 3 and Table 4 present energies of frontier (HOMO, LUMO) orbitals, E_HOMO_ and E_LUMO_ (eV), the band gap for XMo_12_O_40_^n−^ systems in vacuum and in solvents (water and acetonitrile) as well as atomic composition of frontier orbitals, for all studied systems.

The calculated parameters differ quantitatively from those obtained for XW; nevertheless, the changes observed during transitions between groups or within groups are qualitatively identical for both XW and XMo systems. This suggests that the methodology used for XW systems (linear correlation between E_LUMO_/E_int_ and redox potential) can also be apply to the XMo system.

Based on the relationship between E_LUMO_ and charge of the central ion, found for the XW and XMo systems, the dependence of XW vs. XMo was determined. Next, using the correlation between E_LUMO_ vs. redox potential obtained for XW systems in acetonitrile and on the previously figured mathematical relationship between XW and XMo, the predicted redox potential values for XMo systems were found (details are described in the Supplementary Materials). The values are collected in Table 5, where additionally the last line presents the experimentally determined redox potential values taken from the literature [85].

The comparison of the values obtained from the mathematical model and those taken from the experimental measurements clearly shows that the mathematical model works properly, and the predicted redox potential values for most of the central ions are almost identical to the experimental data (the only exception is S, for which the predicted redox potential value is almost twice as high as the measured value).

This allows us to assume that the mutual similarities and the correlations between the theoretical parameters calculated for the XW and XMo systems form the basis for the qualitative and quantitative prediction of redox potential being one of the possible parameters describing catalytic properties.

## 3. Materials and Methods

**Figure 7:**
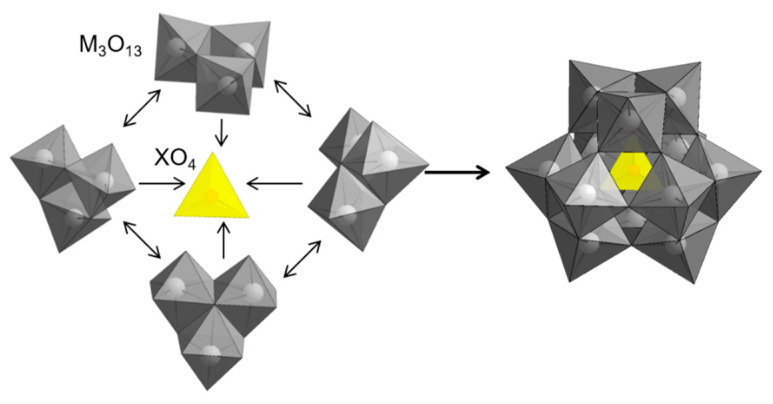
Geometries of building elements of Keggin anion.

One can find many HPA systems with different structures but the most important for catalysis are those containing one central atom/heteroatom and twelve metal/addenda ions; known as Keggin anions described by the general formula Y_n_XM_12_O_40_. In this particular structure, the central position is occupied by ion, X, coordinated to four oxygen atoms, forming internal tetrahedron XO_4_^n−^. Tetrahedron is surrounded by twelve edge- and corner-sharing metal–oxygen octahedral MO_6_, which are arranged in four M_3_O_13_ groups, each formed by three octahedral sharing edges and having common oxygen atom, which is also shared with central XO_4_ tetrahedron (Figure 7).

To model the influence of central ion substitution on the oxidizing power of modified molybdenum and tungsten heteropolyacids, a series of p-block elements were selected; in addition, zinc was also taken into consideration. The main selection criterion was the availability of experimental values of redox potentials obtained for a series of compounds in the same conditions to eliminate other parameters. Electronic structure calculations were performed for XW_12_O_40_^n−^ and XMo_12_O_40_^n−^ Keggin anions modified in position of heteroatom, by various elements X = Zn^2+^, B^3+^, Al^3+^, Ga^3+^, Si^4+^, Ge^4+^, P^5+^, As^5+^, and S^6+^. The examined central ions can be ordered according to several criteria. One of them is to divide central ions according to which row of periodic table they belong to. In such a case, the studied systems can be divided into three groups. In the first group the central ion B^3+^ belongs to II row, in the second group central ion belongs to III row (Al^3+^, Si^4+^, P^5+^, and S^6+^) and in the third group the central ion lies in IV row (Zn^2+^, Ga^3+^, Ge^4+^, and As^5+^). The other possibility is to group central ions according to their formal oxidation state that is equal to formal charge of internal tetrahedron (XO_4_^n−^) and, thus, the global charge of Keggin anion. As a result, one obtains five groups where n = 2 (S^6+^), n = 3 (P^5+^ As^5+^), n = 4 (Si^4+^, Ge^4+^), n = 5 (B^3+^, Al^3+^, and Ga^3+^), and n = 6 (Zn^2+^). However, in our opinion the most proper and adequate solution to the problem is not to divide elements into any group but to take into consideration their position in the periodic table.

The calculations were based on DFT theory and local cluster model. Exchange and correlation energies were calculated using the Perdew–Burke–Ernzenhof functional within the generalized gradient corrected approximation (GGA-PBE) [86] (Turbomole code [87]). The TZVP (basis sets of triple-zeta valence with polarization) base was applied for all elements except transition metal ions (W, Mo) where pseudopotentials were used [88].

Geometry optimizations were performed according to quasi-Newton–Raphsod method within BFGS algorithm proposed by Broyden [89], Fletcher [90], Goldfarb [91], and Shanno [92] independently. Obtained converged geometry satisfies the following parameters: (a) the energy change between two optimization cycles drops below 10^−6^ a.u., (b) the maximum displacement element drops below 10^−3^ a.u., and (c) the maximum gradient element dropped below 10^−3^ a.u.

Different spin states for all systems were checked by performing spin restricted and unrestricted calculations for the close and open shell anions, respectively. In the discussion below only the systems with lowest total energy are discussed, all being singlet states.

The solvation effect was included by COSMO approximation [93] (COnductorlike Screening MOdel) where the solute molecule forms a cavity within the dielectric continuum of permittivity ε (for ideal solvent ε = ∞) that represents the solvent. The cavity construction starts with a union of spheres of radii R_i_ + R_solv_ for all atoms i.

Interaction between internal tetrahedron (XO_4_^n−^) and metal–oxygen framework was performed according to energy decomposition analysis scheme (EDA) developed by Morokuma [94] and by Ziegler and Rauk [95].

## 4. Conclusions

The presented results of calculations show that the type of central ion influences both the energy levels of boundary orbitals and the size of band gap, parameters which determine the oxidation-reduction potential of heteropolyacids systems. The comparison of theoretical and experimental data leads to the conclusion that theoretical energy of LUMO orbitals (obtained for systems in solvent) gives an important information about the redox potential of the system.

Moreover, a total energy interaction (ΔE_int_) can be a proper theoretical parameter to reflect the oxidizing ability of heteropolyanions modified in the central ion position in both tungsten and molybdenum systems.

The analysis of density of states spectra clearly shows that the effect of the central ion on the energy of frontier orbitals of heteropolyacids anions takes place through the various oxygen centers present in the area of the valence energy near the Fermi level, the characteristics of which change along with the type of the central ion. The participation of both the central ion orbitals and the Oa oxygen orbitals directly coordinated with it, in the zone close to the valence level, unequivocally explains the influence of the X ion on the energy of the boundary orbitals and the size of the energy gap.

## Figures and Tables

**Table 1:** Energies of frontier (HOMO, LUMO) orbitals, E_HOMO_ and E_LUMO_ (eV) and the band gap for XW_12_O_40_^n−^ systems in vacuum, water and acetonitrile-CH_3_CN (top, middle, and bottom part of the table, respectively). Additionally total energy, ΔE_int_ (a.u.), and experimental redox potential E_red_ (V) for XW_12_O_40_^n−^ system in acetonitrile, are presented (the last two rows).

X	Zn^2+^	B^3+^	Al^3+^	Ga^3+^	Si^4+^	Ge^4+^	P^5+^	As^5+^	S^6+^
Vacuum									
E_HOMO_	8.23	5.99	5.29	5.28	2.59	2.57	−0.15	−0.16	−2.89
E_LUMO_	10.52	7.99	7.87	7.84	5.17	5.13	2.41	2.37	−0.38
Gap	2.29	2.00	2.58	2.56	2.58	2.56	2.56	2.53	2.51
Water (H_2_O)									
E_HOMO_	−5.78	−5.71	−6.43	−6.43	−6.74	−6.73	−7.04	−7.03	−7.33
E_LUMO_	−3.55	−3.77	−3.83	−3.84	−4.14	−4.16	−4.47	−4.49	−4.81
Gap	2.23	1.94	2.60	2.59	2.60	2.57	2.57	2.54	2.52
Acetonitrile (CH_3_CN)									
E_HOMO_	−5.50	−5.48	−6.20	−6.19	−6.55	−6.55	−6.89	−6.90	−7.24
E_LUMO_	−3.26	−3.53	−3.59	−3.61	−3.95	−3.97	−4.33	−4.36	−4.72
Gap	2.24	1.95	2.61	2.58	2.60	2.58	2.56	2.54	2.52
ΔE_int_ [a.u.]	−1.122	−0.907	−0.808	−0.804	−0.570	−0.571	−0.345	−0.348	−0.145
E_red_ [eV]	−1.92	−1.65	−1.56	−1.54	−1.13	−1.09	−0.64	−0.67	−0.26

ΔE_int_—total energy interaction between the internal tetrahedron (XO_4_^n−^) and the metal–oxygen framework (W_12_O_36_). E_red_—experimentally [84] obtained redox potential.

**Table 2:** Decomposition into atomic contribution (in %) of HOMO and LUMO orbitals in XW_12_O_40_^n−^ system.

X	B^3+^	Al^3+^	Si^4+^	P^5+^	S^6+^	Zn^2+^	Ga^3+^	Ge^4+^	As^5+^
HOMO									
X	0.00	0.01	0.00	0.00	0.00	12.25	0.02	0.00	0.00
Oa	**77.43**	0.39	0.03	0.01	0.01	**50.56**	0.93	0.09	0.02
W	4.16	0.61	0.70	0.84	1.05	2.04	0.64	0.74	0.88
Ob	1.67	**44.78**	**46.47**	**47.26**	**47.71**	2.21	**43.81**	**46.03**	**46.92**
Oc	6.72	**52.08**	**51.46**	**50.88**	**50.49**	**26.01**	**51.84**	**51.56**	**50.99**
Od	**10.03**	2.12	1.34	1.01	0.75	6.94	2.76	1.57	1.19
LUMO									
X	0.00	0.00	0.00	0.00	0.00	0.00	0.00	0.00	0.00
Oa	0.01	0.01	0.00	0.01	0.01	0.01	0.02	0.01	0.00
W	**75.39**	**75.28**	**74.99**	**74.71**	**74.48**	**75.60**	**75.29**	**74.99**	**74.73**
Ob	**11.62**	**11.69**	**11.79**	**11.93**	**12.04**	**11.68**	**11.69**	**11.78**	**11.88**
Oc	**12.80**	**12.89**	**13.08**	**13.23**	**13.35**	**12.61**	**12.91**	**13.11**	**13.28**
Od	0.19	0.15	0.14	0.13	0.11	0.12	0.13	0.13	0.12

**Table 3:** Energies of frontier (HOMO, LUMO) orbitals, E_HOMO_ and E_LUMO_ (eV) and the band gap for XMo_12_O_40_^n−^ systems in vacuum, water, and acetonitrile-CH_3_CN (top, middle, and bottom part of the table, respectively). Additionally total energy, ΔE_int_ (a.u.), for XMo_12_O_40_^n−^ system in acetonitrile, are presented (in last row).

X	Zn^2+^	B^3+^	Al^3+^	Ga^3+^	Si^4+^	Ge^4+^	P^5+^	As^5+^	S^6+^
Vacuum									
E_HOMO_	8.20	6.18	5.47	5.45	2.81	2.78	0.10	0.07	−2.65
E_LUMO_	10.73	8.00	7.96	7.95	5.14	5.13	2.30	2.29	−0.55
Gap	2.53	1.82	2.49	2.50	2.33	2.35	2.20	2.22	2.10
Water (H_2_O)									
E_HOMO_	−5.87	−5.61	−6.32	−6.33	−6.59	−6.61	−6.90	−6.88	−7.19
E_LUMO_	−3.38	−3.84	−3.82	−3.81	−4.25	−4.24	−4.68	−4.67	−5.08
Gap	2.49	1.77	2.50	2.52	2.34	2.37	2.22	2.21	2.11
Acetonitrile (CH_3_CN)									
E_HOMO_	−5.87	−5.37	−6.08	−6.09	−6.40	−6.42	−6.74	−6.76	−7.10
E_LUMO_	−3.38	−3.60	−3.58	−3.57	−4.06	−4.05	−4.54	−4.53	−4.99
Gap	2.48	1.77	2.50	2.52	2.34	2.37	2.20	2.23	2.10
ΔE_int_ (a.u.)	−1.248	−0.900	−0.796	−0.795	−0.660	−0.643	−0.443	−0.448	−0.254

ΔE_int_—total energy interaction between the internal tetrahedron (XO_4_^n−^) and the metal–oxygen framework (Mo_12_O_36_).

**Table 4:** Decomposition into atomic contribution (in %) of HOMO and LUMO orbitals in XMo_12_O_40_^n−^ system.

X	B^3+^	Al^3+^	Si^4+^	P^5+^	S^6+^	Zn^2+^	Ga^3+^	Ge^4+^	As^5+^
HOMO									
X	0.00	0.00	0.00	0.00	0.00	10.82	0.00	0.00	0.00
Oa	**76.12**	0.12	0.02	0.01	0.01	**49.90**	0.23	0.11	0.01
W	5.79	1.65	1.70	1.89	2.19	2.01	1.70	1.74	1.96
Ob	1.89	**41.47**	**43.51**	**44.86**	**45.80**	2.52	**40.82**	**45.47**	**44.25**
Oc	5.82	**55.13**	**53.54**	**52.27**	**51.24**	**24.20**	**55.46**	**51.21**	**52.72**
Od	**10.39**	1.63	1.23	0.98	0.76	10.56	1.78	1.47	1.05
LUMO									
X	0.00	0.00	0.00	0.00	0.00	0.00	0.00	0.00	0.00
Oa	0.06	0.03	0.02	0.01	0.00	0.02	0.02	0.00	0.00
W	**70.29**	**69.74**	**70.10**	**70.34**	70.44	**69.33**	**69.62**	**71.83**	**70.16**
Ob	**14.45**	**15.04**	**14.64**	**14.34**	**14.13**	**15.46**	**15.11**	**12.92**	**14.51**
Oc	**14.14**	**14.14**	**14.44**	**14.73**	**14.99**	**13.99**	**14.19**	**15.01**	**14.72**
Od	1.05	1.05	1.23	0.58	0.44	1.20	1.06	0.27	0.61

**Table 5:** Redox potential appointed using theoretical methods and experimentally obtained [85] redox potentials for molybdenum XMo_12_O_40_^n−^ heteropolyanions in acetonitrile (CH_3_CN).

X.	Al^3+^	Ga^3+^	Si^4+^	Ge^4+^	P^5+^	As^5+^	S^6+^
CH_3_CN							
Model	−1.56	−1.54	−1.10	−1.08	−0.63	−0.64	−0.21
E_red_ (eV)	-	-	−1.10	−1.14	−0.64	−0.66	−0.11

## Data Availability

Data are provided by the authors upon a reasonably request.

## Supplementary Materials

The following supporting information can be downloaded. Figure S1: Correlation between en-ergy of LUMO (Lowest Unoccupied Molecular Orbitals) orbitals, ELUMO (eV), and charge of central ions, X, for XW_12_O_40_^n−^ (XW—blue squares) and XMo_12_O_40_^n−^ (XMo—red squares) systems n = 2–5, in CH3CN as a solution, where the central ions, X = Al^3+^, Si^4+^, P^5+^, S6^+^; Figure S2: Corre-lation between energy of LUMO (Lowest Unoccupied Molecular Orbitals) orbitals, ELUMO (eV), and experimental [85] redox potential [V] for XW_12_O_40_^n−^ (XW) systems n = 2–5, in CH3CN as a solution, where the central ions, where X = Al^3+^, Si^4+^, P^5+^, S^6+^; Table S1: Energy of LUMO (Lowest Occupied Molecular Orbital) orbitals, ELUMO, [eV] for XW12O40^n−^ (XW) and XMo_12_O_40_^n−^ (XMo) systems n = 2–5, in CH3CN as a solution, where X = Al^3+^, Si^4+^, P^5+^, S^6+^ (the central ion belongs to the III period of the periodic table); Table S2: Successive coordinates for XMo_12_O_40_^n−^ (XMo) systems n = 2–5, in CH3CN as a solution, where the central ions, X = Al^3+^, Si^4+^, P^5+^, S^6+^, determined in the mathematical model, from the dependence of straight lines ob-tained for the XW and XMo systems from Figure S1.
