# Supplementary material for: Tungsten and Molybdenum Heteropolyanions with Different Central Ions—Correlation between Theory and Experiment
Source: Molecules. 2021 Dec 29;27(1):187. doi: 10.3390/molecules27010187 (PMC8747034; doi:10.3390/molecules27010187)
Supplement: Supplementary file 1 [file molecules-27-00187-s001.zip › molecules-1524866-supplementary.pdf]

Figure S1. Correlation between energy of LUMO (Lowest Unoccupied Molecular Orbitals) orbitals, E_{LUMO} (eV), and charge of central ions, X, for $XW_{12}O_{40}^{n-}$ (XW- blue squares) and $XMo_{12}O_{40}^{n-}$ (XMo - red squares) systems n=2-5, in CH_3CN as a solution, where the central ions, X = Al^{3+}, Si^{4+}, P^{5+}, S^{6+}.

Data are taken from Table S1.

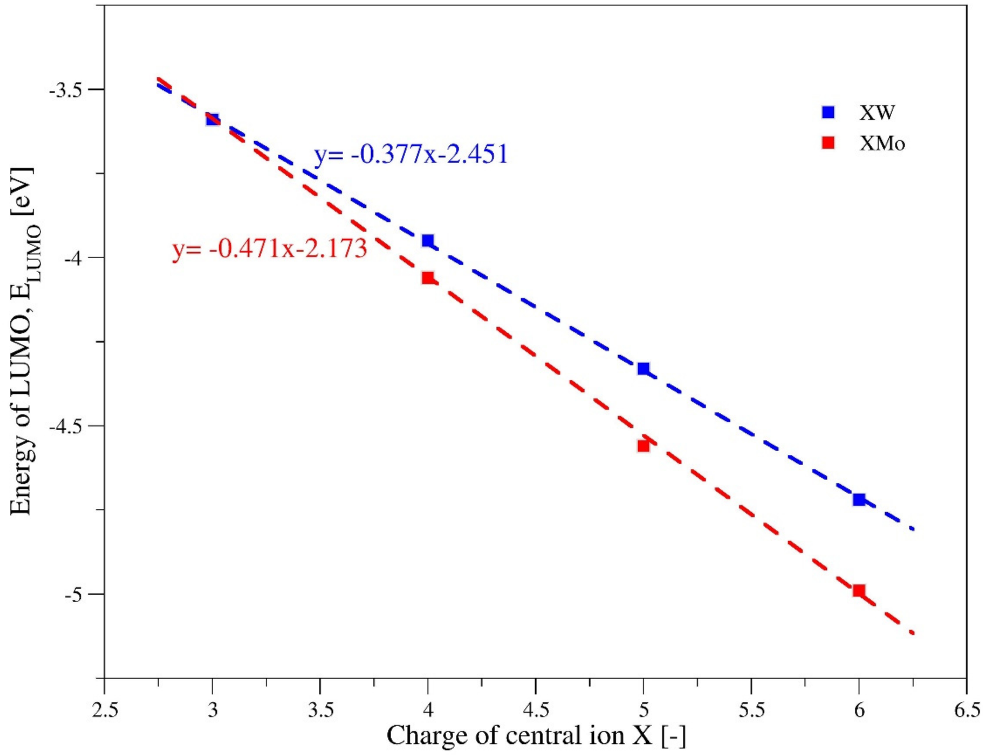

Based on the regression equations (for XW y = -0.377x -2.451 and for XMo y = -0.471x - 2.173) determined:

- coordinates for intersection point P: x_0 = 2.957, y_0= -3.566
- the slope of the regression line for XW: θ_1 = arctg(-0.377) = -20.656°
- the slope of the regression line for XMo: θ_2 = arctg(-0.471) = -25.220°
- the rotational angle between XW and XMo lines: $\theta_3 = \theta_1 - \theta_2$ = 4.564°

Figure S2. Correlation between energy of LUMO (Lowest Unoccupied Molecular Orbitals) orbitals, E_{LUMO} (eV), and experimental [85] redox potential [V] for $XW_{12}O_{40}^{n-}$ (XW) systems n=2-5, in CH_3CN as a solution, where the central ions, where X = Al^{3+}, Si^{4+}, P^{5+}, S^{6+}.

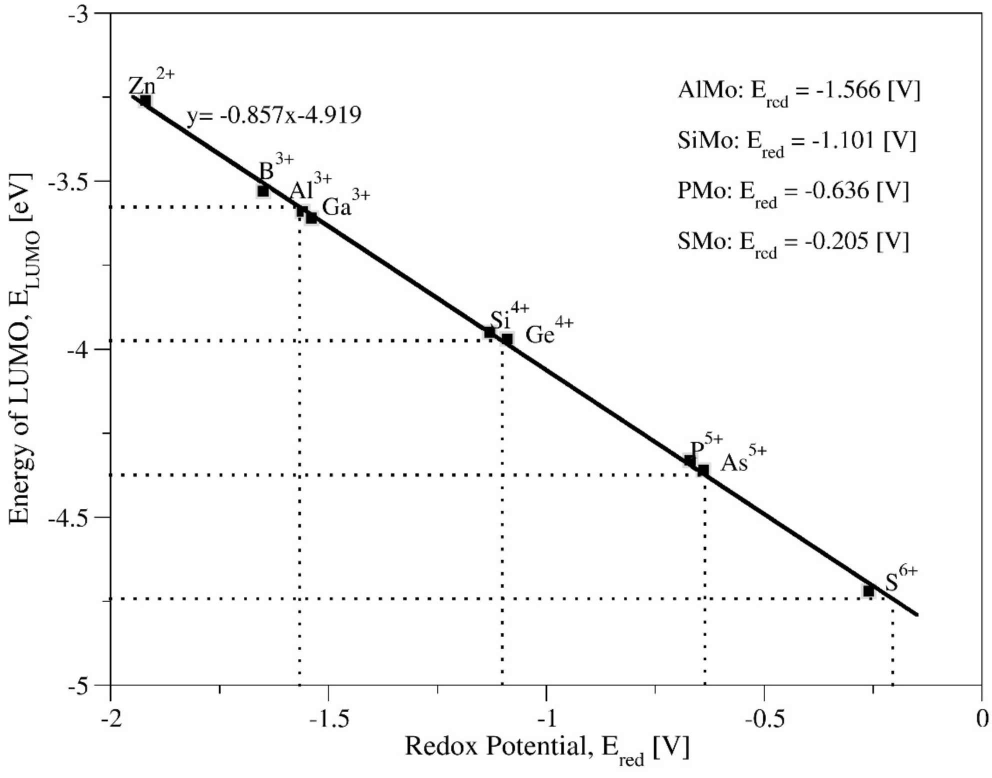

The predicted redox potential for $XMo_{12}O_{40}^{n-}$ (XMo) systems, where X=Al^{3+}, Si^{4+}, P^{5+}, S^{6+} was read from the presented straight line (y = -0.857x - 4.919).

The redox potential was determined for the points, for which the coordinates x' and y' were determined in the mathematical model in Table 2.

Table S1. Energy of LUMO (Lowest Occupied Molecular Orbital) orbitals, E_{LUMO}, [eV] for $XW_{12}O_{40}^{n-}$ (XW) and $XMo_{12}O_{40}^{n-}$ (XMo) systems n=2-5, in CH_3CN as a solution, where X = Al^{3+}, Si^{4+}, P^{5+}, S^{6+} (the central ion belongs to the III period of the periodic table).

X	Al	Si	P	S
Charge of X	3	4	5	6
	XW			
E_{LUMO} [eV]	-3.59	-3.95	-4.33	-4.72
	XMo			
E_{LUMO} [eV]	-3.59	-4.06	-4.54	-4.99

Table S2. Successive coordinates for $XMo_{12}O_{40}^{n-}$ (XMo) systems n=2-5, in CH_3CN as a solution, where the central ions, X = Al^{3+}, Si^{4+}, P^{5+}, S^{6+}, determined in the mathematical model, from the dependence of straight lines obtained for the XW and XMo systems from Figure S1.

X	Al	Si	P	S
x=charge of X	3	4	5	6
y=E_{LUMO} [eV]	-3.590	-4.060	-4.540	-4.990
x_1	0.043	1.043	2.043	3.043
y_1	-0.014	-0.494	-0.974	-1.424
x_1'	0.044	1.079	2.114	3.147
y_1'	-0.011	-0.409	-0.808	-1.177
x'	3.001	4.036	5.071	6.104
y'	-3.577	-3.975	-4.374	-4.743

x, y - initial coordinates (presented for XMo system in Table S1) corresponding x=charge of central ions and y=E_{LUMO}=energy of LUMO orbitals [eV].

x_1, y_1 - coordinates obtained by translation/shift ($x_1 = x - x_0$ and $y_1 = y - y_0$) initial coordinates with respect to the new origin of the coordinate system, designated in Figure S1, point P ($x_0 = 2.957$. $y_0 = -3.566$)

x_1', y_1' – coordinates obtained as a result of construction of transition matrix, based on the angle (θ_3=4.564°) of rotation XW and XMo lines (from Figure S1)

$$\begin{matrix} x_1' \\ y_1' \end{matrix} = \begin{matrix} x_1 \cos\theta_3 - y_1 \sin\theta_3 \\ x_1 \sin\theta_3 + y_1 \cos\theta_3 \end{matrix}$$

x', y' - original coordinate system obtained based on rotation matrix, $x' = x_1' + x_0$ and $y' = y_1' + y_0$
